# 27646 Spinal Control Impairments During Two Clinical Tests of Lower Limb Movement in People with and without Low Back Pain

**DOI:** 10.1017/cts.2021.702

**Published:** 2021-03-30

**Authors:** Stacey Chen, Quenten Hooker, Vanessa Lanier, Linda Van Dillen

**Affiliations:** Washington University of St. Louis

## Abstract

ABSTRACT IMPACT: Our work may be able to impact the examination and clinical decision making of a clinician to identify and target movement impairments to treat people with low back pain. OBJECTIVES/GOALS: Test if the magnitude of spinal control impairments is different in two clinical tests of lower limb movement in people with and without low back pain (LBP). The impairment is defined as the time difference between the start of limb to lumbopelvic motion. Also, test if the magnitude of impairments across tests is associated with LBP intensity and function. METHODS/STUDY POPULATION: 18 controls and 21 people with LBP (51.6% F, 34.5 ±11.5 yrs) participated in a cross-sectional, laboratory-based study. Subjects completed the modified Oswestry Disability Questionnaire (LBP-related functional limitation measure; 0-100%) and the Numeric Pain Rating Scale (LBP intensity; average pain prior 7 days; 0-10) self-report surveys and clinical tests of hip medial and lateral rotation performed in prone. A three-dimensional motion capture system was used to capture angular lumbopelvic and hip motion across time. A 2x2 mixed model ANOVA will be used to examine the effects of group, hip rotation test, and group x hip rotation test. Separate bivariate correlations will be used to quantify the association of magnitude of the impairment to (1) average LBP intensity and (2) LBP-related functional limitation. RESULTS/ANTICIPATED RESULTS: We hypothesize that, compared to healthy controls, people with LBP will display a greater magnitude of impairment across the hip medial and lateral rotation tests. In addition, we hypothesize that the magnitude of the difference in impairment between people with LBP vs controls will be larger during the hip lateral rotation test compared to the hip medial rotation test. Finally, we hypothesize that in people with LBP the magnitude of the impairments across tests will be associated with LBP intensity and LBP-related functional limitation. DISCUSSION/SIGNIFICANCE OF FINDINGS: If our hypotheses are supported, the hip rotation tests would further be recognized as a key part of a clinician’s examination and an important target for treatment of LBP.

